# Finite‐element simulation of in‐plane tear propagation in the dissected aorta: Implications for the propagation mechanism

**DOI:** 10.1002/cnm.3743

**Published:** 2023-06-21

**Authors:** Han Han, Baolei Guo, Peng Gao, Fan Yang, Cuiru Sun, Nicholas A. Hill, Haofei Liu

**Affiliations:** ^1^ Department of Mechanics Tianjin University Tianjin China; ^2^ Department of Vascular Surgery Zhongshan Hospital, Fudan University Shanghai China; ^3^ School of Mathematics and Statistics University of Glasgow Glasgow UK

**Keywords:** aortic dissection, cohesive zone method, computer modeling, finite element, tear propagation

## Abstract

Computer modeling and numerical simulation are essential for understanding the progression of aortic dissection. However, tear propagation has not been properly modeled and simulated. The in‐plane dissection propagation between concentrically distributed elastic lamellae is modeled using the cohesive zone method with a bilinear traction‐separation law. The parameters of cohesive elements are calibrated for the three modes of interfacial damage in the media, enabling quantitative predictions of in‐plane tear propagation. An idealized cylindrical tube‐shaped bilayer thick‐wall model of the aorta is employed to elucidate the influence of geometrical parameters, loading conditions and residual stress on the tear propagation. While the model is capable of replicating that deeper, larger tears are associated with lower critical pressure, new findings include: (i) Larger axial stretch leads to lower critical pressure; (ii) Increased magnitude of residual stress is associated with higher critical pressure; (iii) Pressure difference between true and false lumen alters the critical pressure; (iv) The interfacial damage is mostly opening mode in the axial direction, but shear‐dominated in the circumferential direction; (v) Damage due to the opening mode is associated with smaller fracture energy, which makes it easier to propagate than the shear and the mixed modes. (vi) The deformed shape of the tear, which is related to its geometrical features and loading conditions, modulates the critical pressure via two pathways: (a) deformed shapes are associated with specific modes of damage, which influences the critical pressure, and (b) deformed shapes modulate the critical pressure directly via geometrical constraints.

## Graphical abstract

Deeper, larger and more axially‐extended tears are easier to propagate, while the residual stress and a positive pressure difference between the true and false lumen plays a protective role against the propagation. The readiness of tear propagation is modulated via two pathways: (a) modulated by the mode of damage; (b) modulated by the geometrical constraints.

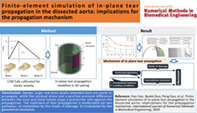

## 1 INTRODUCTION

Aortic dissection (AD) is a cardiovascular catastrophe that seriously threatens human life. AD occurs when blood enters the media through entry tears in the intima. The blood advances anterograde and retrograde by breaking the bonding between concentrically distributed elastic lamellae (referred to as ‘in‐plane’ propagation in this paper). As a result a false lumen (FL) is created, which is a new channel for blood flow. The propagation of the FL in the media tends to reduce blood perfusion to major organs and lead to death. The extent, as well as the direction of the FL propagation, plays an important role in the diagnosis and treatment of AD. For instance, the existence of branch vessels perfused by the FL results in expansion of the false lumen and is a risk factor for adverse of thoracic endovascular aortic repair (TEVAR) outcomes.^1^ Propagation of AD proximally may cause retrograde type A aortic dissection (RTAD), for which surgical management is challenging.^2^, ^3^, ^4^

Carson and Roach^6^ created a dissection in strips of excised porcine upper thoracic aortas and found that the initial tear started to propagate at 77 kPa upon liquid injection. Tam et al.^5^ studied the propagation pressure of a bleb using an in vitro porcine model and found that the pressure inversely correlated with the initial tear depth of intimal flap. Guo et al.^7^ created aortic dissection models in swine in vivo and concluded that deeper tears progressed more easily. Although these phenomena have been reported experimentally, the mechanism of in‐plane propagation of an existing tear in the arterial wall is yet to be revealed.

Computer modeling and numerical simulations are helpful in revealing the mechanism of AD propagation and predicting its occurrence. The cohesive zone model (CZM) has been utilized in a number of studies to simulate delamination between the lamellar units in the media.^8^, ^9^, ^10^, ^11^, ^12^ Wang et al.^13^ used the energy release rate to determine the tear propagation when subject to internal pressure loading. The method was applied in a two‐dimensional (2D) strip model and it was found that tears parallel to fiber directions are associated with decreased energy release rates. FitzGibbon and McGarry^14^ experimentally characterized mode‐II crack initiation and propagation on ovine aorta and calibrated the CZM parameters for mode‐II damage. Wang et al.^15^ developed a computational model of arterial dissection in a two‐layer cylindrical model using the HGO constitutive model and the XFEM approach. Once internally pressurized, the 2D model was able to simulate AD propagation in both the radial and the circumferential directions, showing that a deeper tear with a larger circumferential angle propagates more easily. Wang et al.^16^ studied dissection propagation with a 2D plane‐strain residually stressed two‐layer arterial model and reported that residual stress protected the arterial wall from dissection by increasing the critical pressure. Zhang et al.^17^, ^18^ proposed a three‐dimensional (3D) model of aortic dissection, where high stiffness springs were utilized to model the bonding between media and adventitia. The model was utilized to create the false lumen so that aortic expansions following dissection can be investigated. Ban et al.^19^ utilized a phase‐field finite‐element model to study stepwise progressive tearing and prevalent sudden tearing in a slab‐shaped aortic sample subject to fluid injection loading, and reported that the critical pressure is inversely correlated with the torn area.

Despite the progresses, the model parameters for interfacial damage employed in recent studies were not fully calibrated with experimental data, which limited quantitative prediction of dissection propagation. Moreover, in‐plane tear propagation in both circumferential and axial directions has not been studied in a fully 3D artery model. Finally, the mechanism of in‐plane tear propagation was not addressed. This paper aims to address the above‐mentioned three problems: a computational model is proposed that incorporates the three modes of interfacial damage (one opening and two sliding modes) using the CZM and simulates in‐plane tear propagation in both the circumferential and axial directions with an idealized bi‐layer 3D aortic model. Upon studying the critical pressure and the mode of damage, a possible mechanism for the tear propagation is also proposed.

## 2 MATERIALS AND METHODS

### 2.1 Constitutive laws

The mechanical response of the aortic wall is described with the nearly incompressible fiber‐reinforced hyperelastic HGO model,^9^, ^20^ which is widely used to characterize the material properties of arteries.^21^ The strain energy function is

(1) $$W=C_{10}\left({\bar{I}}_{1}-3\right)+\frac{1}{D}\left[\frac{{\left(J\right)}^{2}-1}{2}-\ln\left(J\right)\right]+\frac{k_{1}}{2k_{2}}\left[\exp\left(k_{2}{\varepsilon_{1}}^{2}\right)-1\right]+\frac{k_{1}}{2k_{2}}\left[\exp\left(k_{2}{\varepsilon_{2}}^{2}\right)-1\right]$$

with

(2) $$\begin{array}{l} \varepsilon_{1}=\kappa \left({\bar{I}}_{1}-3\right)+\left(1-3\kappa \right)\left({\bar{I}}_{4}-1\right)\\ \varepsilon_{2}=\kappa \left({\bar{I}}_{1}-3\right)+\left(1-3\kappa \right)\left({\bar{I}}_{6}-1\right), \end{array}$$

where *C*_10_, *k*_1_, *k*_2_, *κ*, and *D* are material parameters, **F** is the deformation gradient tensor, $J=\det F\left(\approx 1\right)$ for a nearly incompressible material. The invariants ${\bar{I}}_{1}$, ${\bar{I}}_{4}$, and ${\bar{I}}_{6}$ can be written as

(3) $${\bar{I}}_{1}=\mathrm{tr}\left(\bar{C}\right),{\bar{I}}_{4}=\bar{C}:\left(M⊗M\right),{\bar{I}}_{6}=\bar{C}:\left(N⊗N\right),$$

where $\bar{C}=J^{-\frac{2}{3}}C$, $C=F^{T}\cdot F$. **M** = (cos θ, sin θ, 0) and **N** = (cos θ, −sin θ, 0) are the structural tensors that denote the orientations of the two fiber families. *θ* is the angle between the mean fiber direction and the circumferential direction in the referential configuration.

The material parameters for the bulk material are adopted from biaxial testing of a human aortic sample^22^ and shown in Table 1.

**TABLE 1:** Material parameters for the media and adventitia of the human arterial wall.

Parameter	*C*_10_	*k*_1_ (kPa)	*k*_2_	*κ*	*θ* (°)	*D*
Media	23.0097	127.0692	4.4952	0.3201	0.0008	10^−6^
Adventitia	8.2649	71.2311	1.6901	0.3013	0.001	10^−6^

Based on the observations that the media layer consists of concentric lamellar units^23^ and that tears propagate within the media, we assume the dissection propagates to break the bonding between the lamellar units. Therefore, cohesive elements with zero thickness were embedded in the media to simulate the bonding between the lamellae.

**FIGURE 1:**
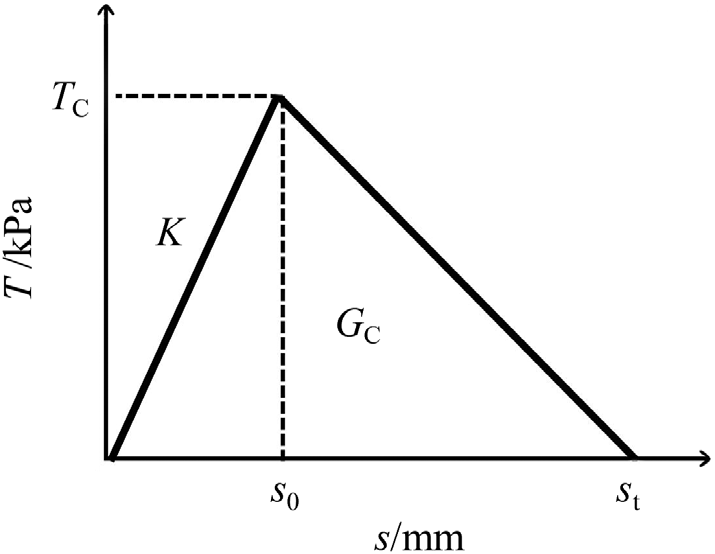
Constitutive relation of the cohesive elements.

Miao et al.^24^ demonstrated that the force‐displacement relationships predicted with the bilinear model matched well with the experiments. Consequently, this study adopts the same model (Figure 1) to characterize the three modes of interface damage, namely, the damage due to the separation force normal to the interface, named the opening mode (Mode‐I), and the damage due to shear force along the interface, called sliding modes (Mode‐II and Mode‐III). The traction *T* in the CZM is taken to be a linear function of the relative separation *S*, before damage initiation occurs at the critical value of the traction *T*_C_. *S*_0_ and *S*_t_ are the separation displacements at damage initiation and complete delamination, respectively, while *G*_C_ represents the critical fracture energy (per unit area) expressible as *G*_C_ = *T*_C_ *S*_t_/2. The initial interfacial stiffness is *K* = *T*_C_/*S*_0_.

The initiation of damage is governed by a quadratic nominal stress criterion,

(4) $${\left\{\frac{\left\langle T_{n}\right\rangle }{T_{\mathrm{nC}}}\right\}}^{2}+{\left\{\frac{T_{s}}{T_{\mathrm{sC}}}\right\}}^{2}+{\left\{\frac{T_{t}}{T_{\mathrm{tC}}}\right\}}^{2}=1,$$

where *T*_*nC*_, *T*_*sC*_, and *T*_*tC*_ are the maximum normal and shear traction values, respectively, to be determined from experiment, whereas *T*_*s*_ and *T*_*t*_ are values of tractions in pure modes II and III, respectively. Here

(5) $$\left\langle T_{n}\right\rangle =\left\{\begin{array}{c} T_{n}\;\\ 0\; \end{array}\right.\begin{array}{c} \text{if}\;\\ \text{if}\; \end{array}\begin{array}{c} T_{n}\geq 0\\ T_{n}<0 \end{array}\;\begin{array}{c} \left(\text{tension}\right)\\ \;\left(\text{compression}\right) \end{array},$$

The power law is also used to define the failure criterion under mixed‐mode loading^1^ is

(6) $${\left\{\frac{G_{Ι}}{G_{ΙC}}\right\}}^{\alpha }+{\left\{\frac{G_{\mathrm{ΙΙ}}}{G_{\mathrm{ΙΙ}C}}\right\}}^{\alpha }+{\left\{\frac{G_{\mathrm{ΙΙΙ}}}{G_{\mathrm{ΙΙΙ}C}}\right\}}^{\alpha }=1,$$

where *G*_I*C*_, *G*_II*C*_, and *G*_III*C*_ are the critical fracture energy values (per unit area) in pure modes I, II and III, respectively, whereas *G*_I_, *G*_II_, and *G*_III_ are the actual fracture energies (per unit area) for the three modes of damage, respectively. The model was found to be insensitive to the material parameter *α* (see Appendix A for details), so it was set to be 1. The cohesive elements are completely damaged when the damage criterion (Equation 6) is satisfied.

### 2.2 Calibration of CZM parameters

In this section, the peeling test, direct tension test and the shear test performed by Sommer et al.^25^ and Sommer et al.,^26^ respectively, are employed to calibrate the CZM parameters of the media: *T*_*nC*_, *T*_*sC*_, *T*_*tC*_ *G*_I*C*_, *G*_II*C*_, *G*_III*C*_.

**FIGURE 2:**
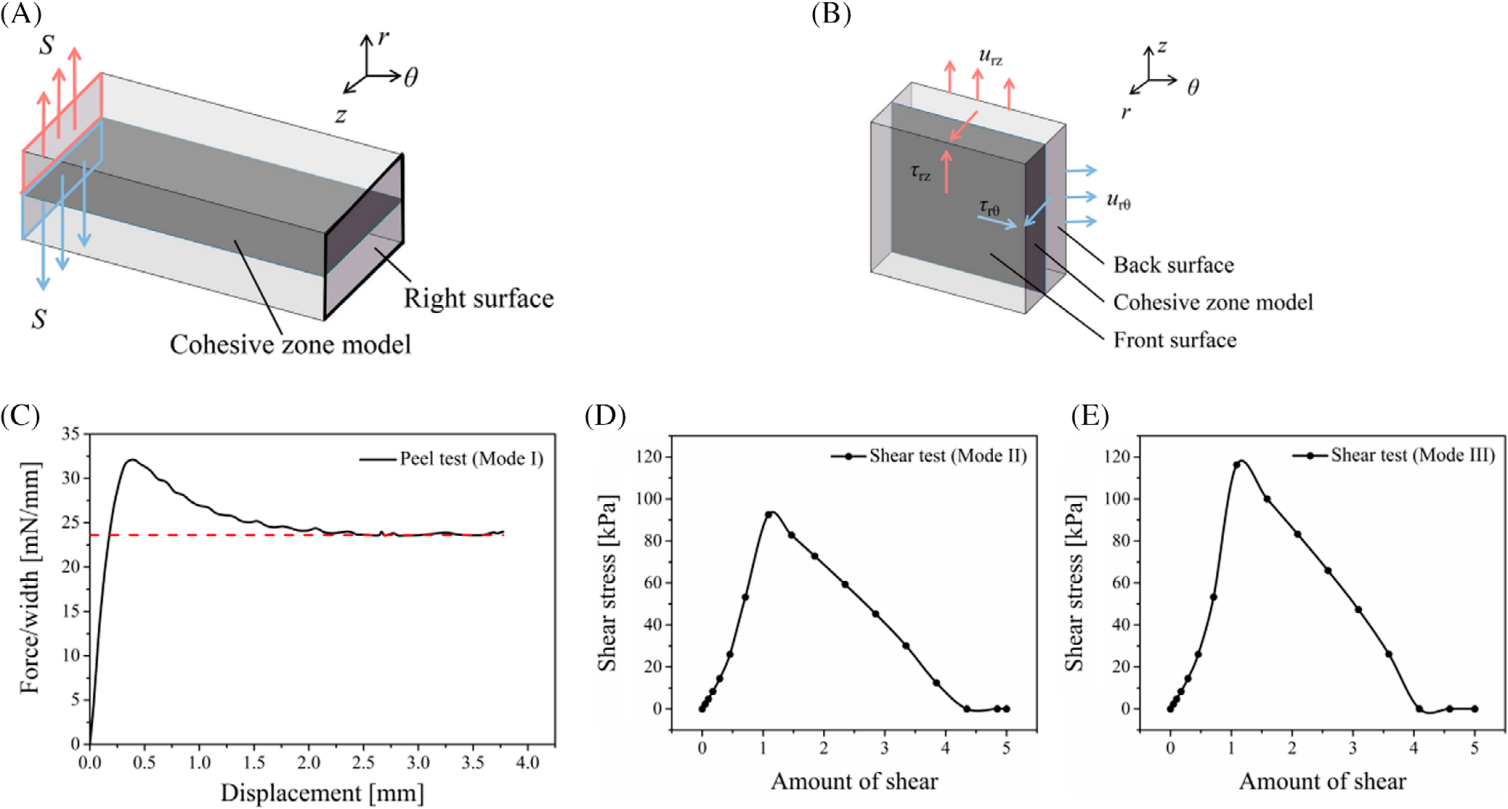
Peeling and shear testing to calibrate the CZM parameters. (A) Sketch of the peeling test. (B) Sketch of the shear test. (C) The relationship between loading per unit width and the displacement during the simulation of peeling test. (D,E) The relationship between Cauchy shear stress and the amount of shear during shear tests in the *rθ*‐ and *rz*‐directions, respectively.

The medial specimen for the peeling test is modeled as a 4.0 mm × 1.2 mm rectangular strip in the *r*–*θ* plane (Figure 2A). Displacement boundary conditions were applied on the left edge in the *r*‐direction to break the cohesive elements and peel the sample apart. The maximum traction for mode‐I damage is taken as *T*_*nC*_ = 131 kPa, according to direct tension tests.^26^ *G*_I*C*_ was adjusted in the simulation until the predicted pulling force per unit width was in agreement with the average (23 mN/mm) from the experiments of Sommer et al.^25^ (Figure 2C **)**. As a result, *G*_I*C*_ = 49 N/m was selected for the model.

To calibrate parameters of the sliding mode, the specimen for shear test is modeled as a 4 mm × 4 mm rectangular strip in *z*–*θ* plane and 1 mm in the radial direction to agree with the experimental setup^26^ (Figure 2B). While the front surface of the specimen was fixed to avoid rigid body motion, the displacement boundary condition is applied on the rear surface in the circumferential and axial directions respectively. The maximum shear stress of modes II and III measured in the experiment were selected as the tractions at damage initiation, *T*_*sC*_ and *T*_*tC*_, respectively. *G*_II*C*_ and *G*_III*C*_, which are essentially the area enclosed by the curve of shear stress versus amount of shear, were determined so that the separation displacement at complete damage was in agreement with the experiment. (Figure 2D,E). The stiffness K was found to have little effect on the simulation results; see Appendix B for details. The calibrated parameters of the CZM are shown in Table 2.

**TABLE 2:** Material parameters for the cohesive elements.

Parameter	*T*_*C*_ (kPa)	*G*_*C*_ (N/m)	*K* (mN/mm^3^)
Mode I (Opening)	131	49	1638
Mode II (Sliding)	97	200	35,000
Mode III (Sliding)	120	240	35,000

### 2.3 Model setup

**FIGURE 3:**
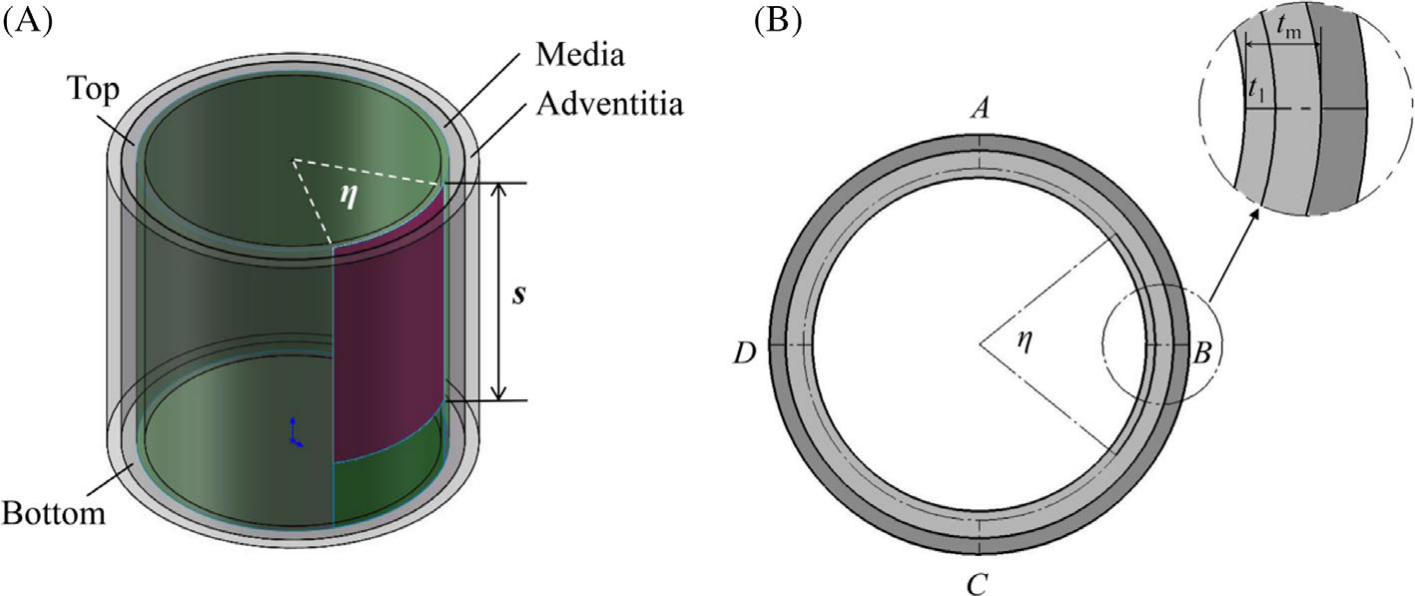
The bi‐layer idealized model of the aortic wall. (A) The three‐dimensional model. (B) The cross‐section of the model.

Dissections take place suddenly and the tears already exist when the patients arrive at the hospital. It is therefore of clinical interest to study the tear propagation at a specific stage of dissection with an initial tear. While the dissections always originate from aortic arch with large curvatures, it is common that the tear propagates distally in the descending aorta that is much less curved than the aortic arc. Therefore, the human descending aorta is idealized as a bilayer cylindrical tube with the inner radius *R*_*i*_ = 7.5 mm and outer radius *R*_0_ = 9 mm with the media/adventitia thickness ratio of 5:3.^27^ While cohesive elements with zero thickness were embedded into the media to simulate the bonding between the lamellae (green zone in Figure 3A), an initial torn region was created (purple zone in Figure 3A). The initial tear was characterized with a central angle *η*, an axial length *s* and a dimensionless depth ratio *t = t*_*1*_ */t*_*m*_. *t*_*m*_ is the thickness of media, while *t*_1_ is the distance between the initial tear and the lumen. The axial length of the aortic wall was set to be 3 mm longer than the initial tear, which was shown to be sufficient to exclude the influence of boundary conditions; see Appendix C for details. C_3_D_8_H and COH_3_D_8_ elements were utilized to discretize the aortic bulk material and interface, respectively. Mesh independence tests were performed (see Appendix E for details). The boundary conditions are set as follows. The axial direction of the bottom face is constrained, while the nodes on the cross‐section A, B, C and D are fixed in the circumferential direction (Figure 3). In particular, friction‐free hard contact was set on both sides of the cohesive elements to prevent penetration.

To simulate the in vivo condition, an axial stretch *λ* was imposed on the top surface (Figure 3A). The same pressure was applied on both the true and the false lumen that is, the initial torn regions and was gradually increased until the first cohesive element was completely damaged and removed from the model, at which moment the pressure is taken to be the critical pressure *P*_*c*_. Additionally, the mode mix ratio, *m*, was defined to indicate the mode of damage taking place during dissection propagation:

(7) $$m=1-\frac{G_{I}}{G_{T}},$$

where *G*_*T*_ *= G*_I_ *+ G*_II_ *+ G*_III_ is the total fracture energy. When *m* = 0, the damage is a pure opening mode, while it is a pure sliding mode if *m* = 1.

The constitutive models, as well as the computational model introduced above, were implemented in ABAQUS/Standard 6.14 (Dassault Systèmes, RI, USA) for static solutions.

## 3 RESULTS

### 3.1 Verification

The damage criteria, as well as the calibrated CZM parameters are employed in this section to replicate the experiment by Tam et al.^5^ for validation purposes. In the experiment, elliptical blebs were created by injection in media of porcine thoracic wall. The blebs were 25–30 mm in length and nearly 180° in circumferential angle. Circumferential slits were made to connect TL with FL, so that the pressure was maintained identical at both lumens. Critical pressures were recorded for blebs of different depth when the blebs began to propagate. Therefore, a section of thoracic aorta was numerically constructed and an initial tear of similar size (*s* = 25 mm, *η* = 180° and *t* = 0.6) was made to simulate the initial bleb in the experiment. Details about the numerical setups can be found in Section 2.3. The predicted critical pressure is 52 kPa, which is well within the range (29.4–63 kPa) reported in experiments. It is worth noting that the critical pressure is closely related to the selection of bulk material parameters. For instance, the bulk material properties extracted from the uniaxial testing data of human thoracic aorta,^28^ along with the CZM parameters calibrated from it, led to a much larger critical pressure of 180 kPa; see Appendix D for details.

### 3.2 Critical pressure

In this section, the initial tear, the axial stretch, as well as the residual stress are investigated for their influence on the critical pressure for dissection propagation. Following Wang et al.,^16^ the values of *η* were chosen to be 90°, 150°, 210°. Since shallow dissections lead to transmural propagation,^15^ *t* = 0.2, 0.4, 0.6 were chosen to prevent it. In addition, *s* was selected to be 5, 10, 15, and 20 mm. The baseline model is chosen to be: *η* = 150°, *t* = 0.4, *s* = 10 mm to facilitate the parametric study.

**FIGURE 4:**
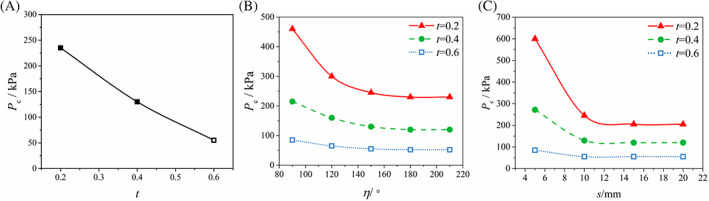
The relationship between the critical pressure and the initial tear. (A) The critical pressure is plotted against the depth ratio. (B) The critical pressure is plotted against the central angle. (C) The critical pressure is plotted against the axial length. The hollow symbols in the key indicate the dissection propagates in the axial direction and the solid symbols indicate the dissection propagates in the circumferential direction.

It is found from Figure 4A that, for an initial tear size *η* = 150°, *s* = 10 mm, the critical pressure decreases monotonically with *t*, that is, deeper dissections are more likely to propagate. Interestingly, as the depth ratio increases, the propagation direction switches from the circumferential to the axial direction, revealing a possible competition in propagation directions.

For an initial dissection *t* = 0.4, *s* = 10 mm, Figure 4B shows that the critical pressure decreases with the central angle *η*. However, *P*_*c*_ is constant when *η* > 180°. Similar trends are observed in the simulations for *t* = 0.2 and 0.6.

For an initial dissection *η* = 150°, *t* = 0.4, the critical pressure decreases with the axial length *s*, and then remains unchanged when *s* > 15 mm. Similar trends are observed in simulations for *t* = 0.2 and 0.6.

Axial stretches of 1.02, 1.22, and 1.42 were investigated for their influence on the critical pressure. From Table 3, it is seen that the larger the axial stretch, the smaller the critical pressure that is, the dissection propagates more easily when the aorta is loaded with larger axial stretches.

**TABLE 3:** The influence of axial stretch on the critical pressure of the baseline model.

Axial stretch	Critical pressure (kPa)
1.02	130
1.22	125
1.42	120

**FIGURE 5:**
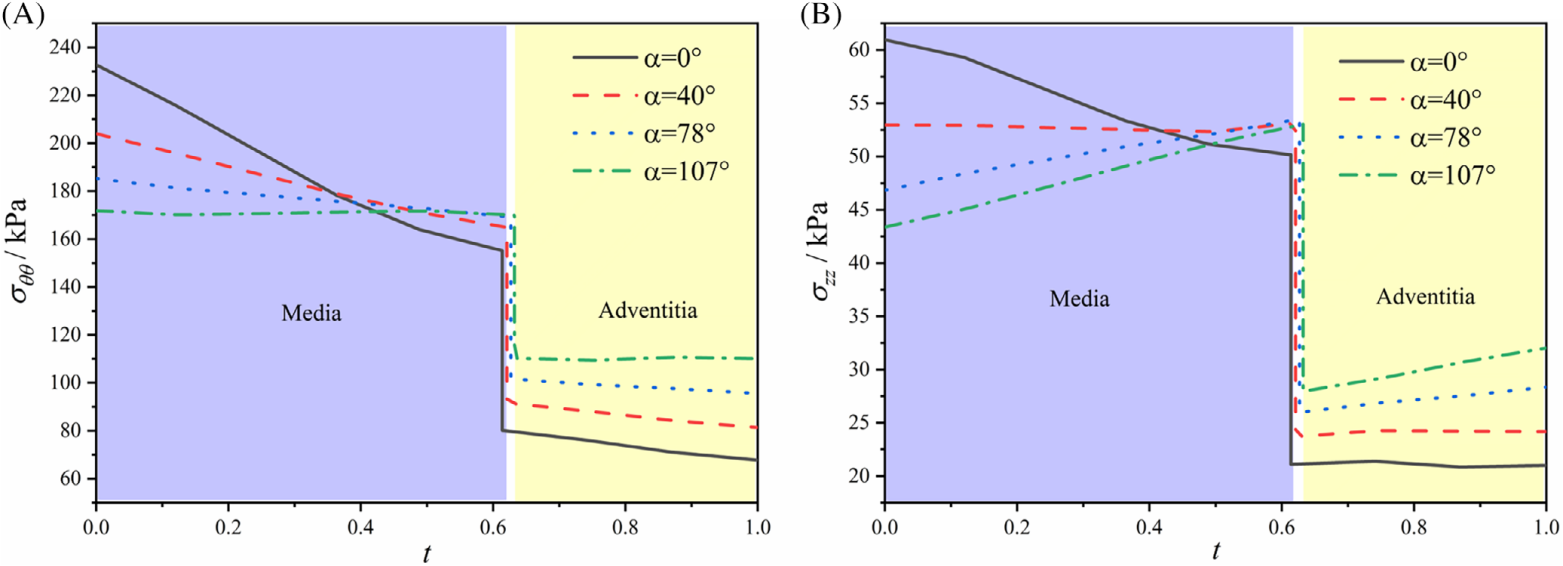
In vivo transmural stress distribution with residual stress (opening angle *α* = 40°, 78°, and 107°) and without residual stress (opening angle *α* = 0°). (A) Circumferential stress and (B) axial stress

The residual stress field is applied to the baseline model to study its influence on dissection propagation. Following,^17^ a 3D residual stress field was implemented as follows: The aortic wall was loaded to its in vivo configuration with axial stretch of 1.02 and average blood pressure of 12.8 kPa. A stress‐mediated anisotropic growth model, which was a kinematic volumetric growth model and implemented via a user‐defined material subroutine of UMAT in ABAQUS (6.14), was then activated to alter the transmural stress gradient, so that the residual stress field can be incorporated naturally. Finally, the desired residual stress field was achieved and it was demonstrable with the numerically simulated opening angle. In vivo transmural stress distribution with and without residual stress was shown Figure 5. In this paper, the residual stress fields, corresponding to opening angles of 0°, 40°, 78° and 107°, were employed to study its influence on the critical pressure.

It is shown in Table 4 that the residual stress tends to increase the critical pressure that is the residual stress plays a protective role in the 3D tear propagation of aortic dissection.

**TABLE 4:** The influence of residual stress on the critical pressure of the baseline model.

Opening angle	Critical pressure (kPa)
0°	130
40°	160
78°	180
107°	200

Finally, the pressure difference between true and false lumens (T/F luminal pressure difference, T/F LPD) was investigated for its influence on the critical pressure. It is shown in Table 5 that, in case of positive T/F LPD, the critical pressure (in the TL) is increased from that when the blood pressure is identical in TL and FL. On the other hand, in case of negative T/F LPD, the critical pressure (in the TL) is less than that when the blood pressure is identical in TL and FL.

**TABLE 5:** The influence of T/F luminal pressure difference (LPD) on the critical pressure of the baseline model.

True lumen pressure (kPa)	False lumen pressure (kPa)	T/F LPD (kPa)
100	119	−19
130	130	0
150	142	8

### 3.3 Mode of damage

**FIGURE 6:**
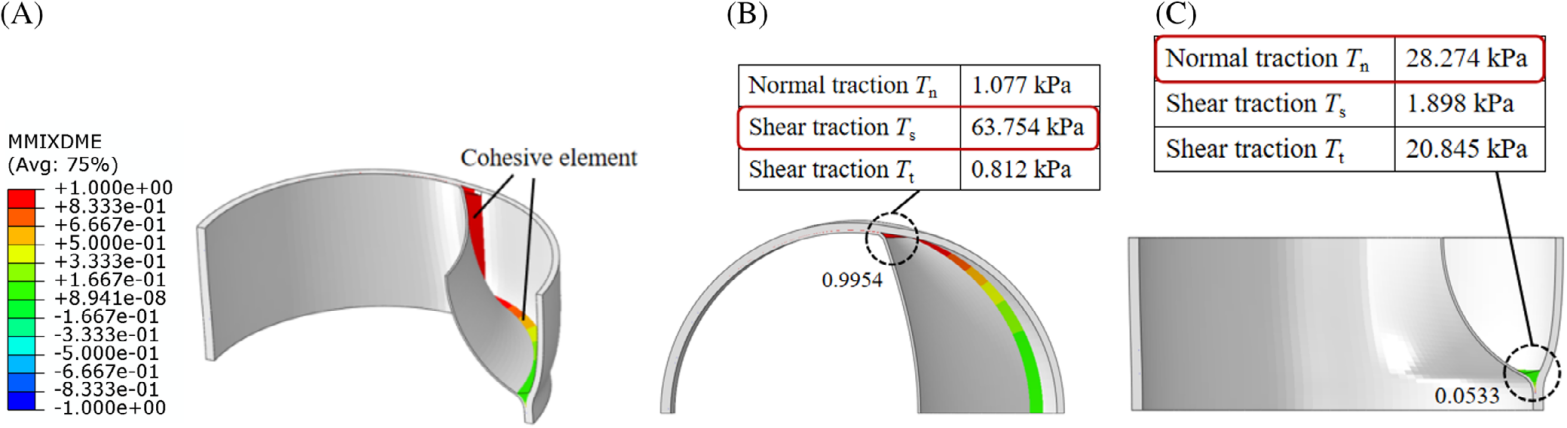
(A) The distribution of mode mix ratio (*m*) for the baseline model. The values of the first cohesive element in the circumferential and axial direction are shown in (B,C). (B) cross‐section of ‘*rθ*–plane’ and (C) cross‐section of ‘rz‐plane’

**FIGURE 7:**
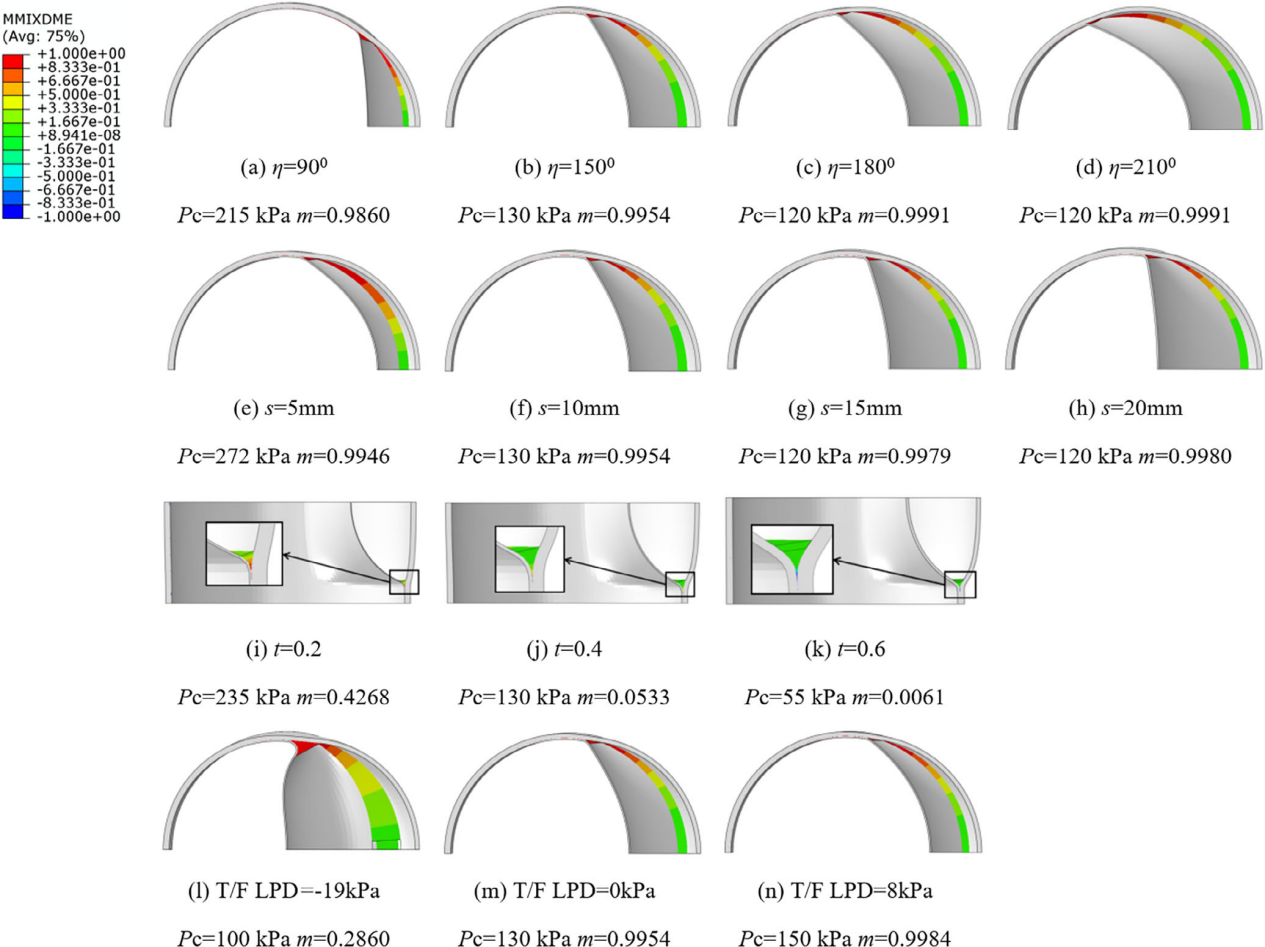
Distribution of the mode mix ratio of the cohesive elements in the cross‐sections of the deformed arterial wall.

In the baseline model (Figure 6 **)**, the mode mix ratio of the first cohesive element in the axial direction equals 0.0533, indicating that the damage in the axial direction is mainly opening mode. Correspondingly, the normal traction of the cohesive element is 28.274 kPa, which is the largest among the three traction forces. The mode mix ratio of the first cohesive element in the circumferential direction is 0.9954, indicating that sliding is the dominant damage mode in this direction. Correspondingly, a shear traction of the cohesive element is 63.754 kPa, which is the largest among the three traction forces. Note that, only the mode mix ratio of the cohesive elements that were in critical states are presented. The above findings are valid too for the cases with different axial stretch and when the residual stress field is incorporated. However, as the T/F LPD transits from negative to positive, the mode mix ratio increases significantly from 0.2860 to 0.9984 that is, the damage is increasingly shear‐dominant (Figure 7L,N **)**.

From Figure 7I–K *m* decreases from 0.4268 to 0.0061 as the depth ratio increases from 0.2 to 0.6 that is, the damage in the axial direction is increasingly dominated by the opening mode for deeper tears. Given the fact that the critical pressure decreases for deeper tears and the fracture energy required to drive the propagation under a pure opening mode is less than that under shear modes, we conclude that the tear propagates more readily under pure mode‐I damage than the mixed mode.

Figure 7A–D shows that, as the central angle increases from 90° to 210°, *m* increases slightly from 0.9860 to 0.9991, thus the damage in the circumferential direction is increasingly pure‐shear‐dominated for longer tears. It is worth noting that when *η* > 180°, the damage mode is almost pure sliding, with the critical pressure remaining constant.

It is noted in Figure 7E–H that as the axial length of the initial dissection increases from 5 to 20 mm, *m* increases from 0.9946 to 0.9980 that is, the damage in the circumferential direction is mainly pure shear for longer tears in the axial direction. In particular, when *s* > 15 mm, the deformed shape of the aortic wall is approximately unchanged, the intimal flap remains vertical and *m* of the cohesive element is almost constant, which coincides with the critical pressure being constant.

### 3.4 Mechanism of dissection propagation

Based on the results of critical pressure and mode of damage, a mechanism for the in‐plane AD propagation can be proposed. Geometrical features of the initial tear (e.g., position and size of the tear) and the loading conditions (e.g., T/F luminal pressure difference) determine the deformed geometry of the arterial wall. On the one hand, the deformed geometry might modulate the critical pressure directly via geometrical constraints. For instance, the constraint to the intimal flap motion, due to small central angle *η* and short axial length *s*, makes it harder to break the bonding between cohesive elements leading to elevated critical pressure. On the other hand, the deformed geometry results in a specific state of traction at the tear, which leads to a specific composition of fracture energies and a specific mode of damage. It is worth noting that the damage mode has a part to play in determining the critical pressure: the fracture energy required to drive the propagation under a pure opening damage mode is less than that under a shear mode (mixed modes in the middle), so that the critical pressure is the lowest for the pure opening mode and highest for the pure shearing modes. However, the geometrical constraint is not significantly altered by the depth ratio of initial tear, axial stretch, residual stress or T/L LPD. Therefore, the critical pressure is correlated to the mode mix ratio *m*: larger *m* (a purer shear mode) corresponds to higher critical pressure.

## 4 DISCUSSION

Although the initiation of aortic dissection is important and has attracted intensive investigations,^29^, ^30^, ^31^ the propagation of aortic dissection is also of clinical interest to understand the progression of the dissection: the direction and the extent of the tear propagation are significant for clinical outcome and treatment strategy. Aortic dissection takes place suddenly, computer modeling and numerical simulation are essential to replicate the propagation process and disclose the mechanism of AD propagation. The partially propagated model in this study was proposed so as to simulate the tear propagation at a specific stage of the lesion development and disclose the underlying mechanism of the tear propagation. It is also worth noting that tear propagation can take place after TEVAR treatment via stent induced new entry tears,^2^, ^32^ where the false lumen already exists and its potential propagation after stent deployment is important to estimate the risks and benefits of the treatment. The partially propagated model in the study paves way for this clinical scenario too.

Peeling and direct tension tests have been employed to calibrate the parameters of interfacial damage.^12^, ^33^, ^34^, ^35^, ^36^ calibrated two separate sets of parameters to simulate the mixed‐mode and pure mode I delamination, respectively. In this paper, published shear test data was employed along with peeling and direct tension tests, so that the opening and sliding parameters were calibrated properly. Moreover, a power law incorporated individual damage modes to enable mixed‐mode damage modeling. Validated against published experimental data, the calibrated CZM model is demonstrated to be capable of simulating the anisotropic interfacial damage of the aortic wall, which leads to quantitative prediction of in‐plane tear propagation.

The critical pressure was found to decrease with the depth of the initial dissection. This agrees well with the experiments by Guo et al.^6^: deeper intimal tears progress more notably. Wang et al.^16^ found that the residual stress elevated the critical pressure in a 2D tear propagation model. This study confirmed the finding in 3D tear propagation settings. Ban et al.^19^ noted that the tearing pressure to be inversely correlated with torn area using a slab‐shaped aortic model. Our study confirms, in a 3D setting of in‐plane tear propagation, that the critical pressure is lowered with the increasing size of initial tear. However, one should not go further to assume the tear would propagate without limit, since the possible crack‐tip blunting effect predicted in FitzGibbon, Fereidoonnezhad, and McGarry,^37^ as well as the influence from bifurcation arteries, were not considered in this study. While the critical pressure was shown to be correlated to the tissue stiffness and the critical energy of tearing by Ban et al.,^19^ our paper highlights the influence from the mode of damage. It is also noted that some of the critical pressures reported in the study are higher than 100 kPa, which are too high for physiological conditions. The reason is twofolds: Firstly, majority of the results were obtained with zero pressure difference between true and false lumen (T/F LPD = 0 kPa). The critical pressure has been shown to be sensitive to T/F LPD: the critical pressure tends to increase as the T/F LPD becomes larger. That is, the critical pressure under zero T/F LPD will be higher than that in cases where T/F LPD is negative, which is frequently observed clinically.^38^, ^39^, ^40^, ^41^ Secondly, high critical pressure was associated with tears of small depth ratio, such as, *t* = 0.2 and *t* = 0.4. The critical pressure was shown sensitive to the depth ratio of the tear in the media: the critical pressure keeps decreasing as the tear moves deeper in the media and it was shown to be as low as 50 kPa when the depth ratio is 0.6. As disclosed in Mitsui et al.,^42^ most of the tears locate at the outer 1/3 in the media (i.e., *t* = 0.67), it is thus expected from our model that the critical pressure would be most frequently seen below 50 kPa. To sum up, high critical pressures reported in the paper are due to the zero pressure difference between true and false lumen, as well as small depth ratio adopted in the baseline model. In the future, fluid–structure‐interaction (FSI) models are needed to calculate the pressure difference between FL and TL, so as to quantify the critical pressure with improved accuracy.

Ban et al.^19^ found that the tear propagation in a slab‐shaped model was of opening mode. Via ex vivo inflation test of bovine aorta, Haslach Jr et al.^30^ reported that the dissection propagation is an in‐plane shear driven process and the damage in the circumferential direction was of mode‐II. With the help of the 3D computational model incorporating all the three damage modes, it was confirmed that the damage mode in the circumferential direction is shear dominated, but also the damage in the axial direction is mainly opening mode.

This study identified that that pure opening mode of damage propagates more easily than pure shear or mixed mode damage and this was partly supported by Leng et al.^12^ who reported that the fracture energy for the mixed‐mode strip is larger than that for the mode‐I strip both in the circumferential and axial direction. It was also confirmed by FitzGibbon and McGarry^14^ that the mode II fracture strength along the circumferential‐axial plane is eight times higher than the corresponding mode I strength. Furthermore, this paper sheds light on the mechanism of in‐plane tear propagation, clarifying the relationships between the geometry of the AD, loading condition, mode of damage and critical pressure.

Although there is a difference in the properties of mode‐I damage between the circumferential and the axial strips, the difference was found not statistically significant.^12^, ^25^ Consequently, the difference in mode‐I properties was not considered in this paper. Recently, Wang et al.^21^ found that axial peeling exhibits a higher energy release rate and strength than circumferential peeling due to the recruitment of both elastin and collagen fibers. The difference might need to be included in future studies. The material parameters for bulk material and interface bonding were not taken from the same sample, therefore the mismatch in material properties may introduce quantitative errors in the results. However, the material properties employed in this study are all from human thoracic aorta, so the qualitative results reported in the paper are not expected to be altered significantly. At last, the straight tube model of aorta ignores the influence of vessel curvatures. While the dissections always originate from aortic arch with a large curvature, it is common that the tear propagates distally in the descending aorta that is much less curved than the aortic arc. It is therefore important to note that the results and conclusions from this study can be more appreciated in the tear progression in the descending aorta. The curvature may have a part to play in much curved aortic arc and ascending aorta which deserves further investigation.

## 5 CONCLUSION

In this paper, we presented a computational model to quantitatively predict three‐dimensional in‐plane propagation of aortic dissection based on a cohesive zone model, in which the parameters of modes I, II, and III damage were calibrated from experimental data. The model was employed to reveal that deeper, larger and more axially extended tears propagate more easily, while the residual stress plays a protective role against AD propagation. Finally, deformed shape of initial tear, which is related to its geometrical features and loading conditions, modulates the critical pressure via two pathways: (a) the deformed shape is associated with specific modes of damage, which will influence the critical pressure; (b) the deformed shape modulates the critical pressure directly via geometrical constraints. This is proposed as the mechanism behind the 3D in‐plane tear propagation.

## CONFLICT OF INTEREST STATEMENT

The authors declare no conflict of interest.

## Data Availability

Data sharing not applicable to this article as no datasets were generated or analysed during the current study.

## APPENDIX A

### A.1

Different values of *α* (0.8, 1.0, 1.5, and 2) were investigated for its influence on the critical pressure using the baseline model (*η* = 150°, *t* = 0.4, *s* = 10 mm). The results show that the critical pressure is 130 kPa which is not affected by *α*.

**TABLE A1:** The parameters of bulk material and cohesive zone method.

Bulk material (media)	*C*_10_ (kPa)	*k*_1_ (kPa)	*k*_2_	*κ*	*θ* (°)	*D*
	13.7	37.4	33.3471	0.3036	7.2321	10^−6^
Interface damage model	*T*_*nC*_ (kPa)	*G*_I*C*_ (N/m)	*T*_*sC*_ (kPa)	*G*_II*C*_ (N/m)	*T*_*tC*_ (kPa)	*G*_III*C*_ (N/m)
	131	52	97	200	120	240

## APPENDIX B

### B.1

When *K*_*n*_ was taken as: 35, 100, 150, 25, 1000, and 1638 mN/mm^3^, the peeling force/width ratios turned out to be 61, 32, 27, 24, 23, and 23 mN/mm, respectively. Therefore, when *K*_*n*_>250 mN/mm^3^, *K*_*n*_ had very little effect on the results, which was in agreement with Leng et al.^10^ and Turon et al.^43^ Since the separation displacement at damage initiation is 80 μm^21^ and the damage initiation stress is 131 kPa for aortic media,^26^ *K*_*n*_ = 1638 mN/mm^3^ is set in this paper. As in the peeling tests, the stiffnesses *K*_*s*_, *K*_*t*_ were found to have very little effect on the results for values greater than 10000 mN/mm^3^ and *K*_*s*_ = *K*_*t*_ = 35000 mN/mm^3^ are used in the paper.

## APPENDIX C

### C.1

Different axial lengths (11, 12, 13, 18, and 23 mm) were set for the baseline model (*η* = 150°, *t* = 0.4, *s* = 10 mm) to investigate the influence of the boundary condition on dissection propagation, corresponding to an axial margin of 1, 2, 3, 8, and 13 mm for the initial tear. While the propagation took place in a different location near the axial constraint for 1 mm margin, the critical pressure was 79, 130, 130, 130 kPa for 2, 3, 8, and 13 mm margin respectively. Since the critical pressure did not change when the margin was longer than 3 mm, the model with axial length of 13 mm was chosen for the simulations.

## APPENDIX D

### D.1

The aortic media parameters were fitted from a uniaxial tensile test^28^ and the cohesive zone method parameters were calibrated from it based on the same peeling test data (Table A1). The aortic adventitia parameters are the same as those in the text. The same model was established as in the Verification section. The critical pressure of the simulation is 180 kPa, compared with the experimental observation of 60 kPa.

## APPENDIX E

### E.1

Mesh independence test was conducted on four sets of meshes: 7920, 13104, 21996, and 41188 elements, respectively. The critical pressure of the baseline model was predicted to be 127, 129, 130, and 130 kPa using the four sets of meshes. As a result, the meshes with 21996 elements was selected for the study.
